# Computational fluid dynamics (CFD) simulation analysis on retinal gas cover rates using computational eye models

**DOI:** 10.1038/s41598-021-84574-2

**Published:** 2021-03-02

**Authors:** Makoto Gozawa, Yoshihiro Takamura, Tomoe Aoki, Kentaro Iwasaki, Masaru Inatani

**Affiliations:** Department of Ophthalmology, Faculty of Medical Sciences, University of Fukui, 23-3 Shimoaizuki, Matsuoka, Eiheiji, Yoshida, Fukui 910-1193 Japan

**Keywords:** Diseases, Medical research

## Abstract

We investigated the change in the retinal gas cover rates due to intraocular gas volume and positions using computational eye models and demonstrated the appropriate position after pars plana vitrectomy (PPV) with gas tamponade for rhegmatogenous retinal detachments (RRDs). Computational fluid dynamic (CFD) software was used to calculate the retinal wall wettability of a computational pseudophakic eye models using fluid analysis. The model utilized different gas volumes from 10 to 90%, in increments of 10% to the vitreous cavity in the supine, sitting, lateral, prone with closed eyes, and prone positions. Then, the gas cover rates of the retina were measured in each quadrant. When breaks are limited to the inferior retina anterior to the equator or multiple breaks are observed in two or more quadrants anterior to the equator, supine position maintained 100% gas cover rates in all breaks for the longest duration compared with other positions. When breaks are limited to either superior, nasal, or temporal retina, sitting, lower temporal, and lower nasal position were maintained at 100% gas cover rates for the longest duration, respectively. Our results may contribute to better surgical outcomes of RRDs and a reduction in the duration of the postoperative prone position.

## Introduction

In recent years, pars plana vitrectomy (PPV) with gas tamponade has become the standard treatment for rhegmatogenous retinal detachments (RRDs)^1–3^. The principle of gas tamponade is that the gas closes the retinal beaks and prevents the vitreous fluid from entering through breaks into the subretinal space^4,5^. Therefore, a proper postoperative position should be maintained after PPV with gas tamponade so that the gas can adequately cover the retinal breaks until the retina is well attached.

The postoperative position after PPV with gas tamponade, particularly for inferior breaks, remains controversial. At present, a strict prone position is recommended^6–11^ to prevent postoperative complications, such as pupillary block, intraocular lens (IOL) dislocation/iris capture, and IOL/cornea touch. However, some patients may experience difficulty in maintaining a strict prone position for medical or physical conditions^12–14^. In addition, Martinez-Castillo et al. reported that PPV alone, with no prone position in the postoperative period, achieves a high reattachment rate without severe postoperative complications in the management of pseudophakic RRDs due to inferior retinal breaks^15^. We have previously demonstrated using magnetic resonance imaging that intraocular gas may cover the peripheral retina better in the supine position than in the prone position^16^. However, sitting and lateral positions are also used postoperatively. In addition, intraocular gas decreases with time after surgery, and the intraocular gas makes the contact angle to the retina that affects retinal gas coverage^5^. Therefore, determining how much area the gas covers in the target retina after PPV with gas tamponade is difficult, and no previous reports have examined changes in retinal gas coverage based on the intraocular gas volume and positions.

Computational fluid dynamic (CFD) analysis is suitable to observe the distribution of gas and fluid considering the physical properties, such as contact angles and surface tensions. Angunawela et al. reported on fluid shear stresses using CFD software on the retinal wall in a model eye after vitrectomy and gas tamponade in relation to eye and head movements^17^. However, no previous report has examined changes in retinal gas coverage based on the intraocular gas volume and positions using fluid analysis. Therefore, this study aimed to investigate how the retinal gas coverage changes due to the intraocular gas volume and positions using fluid analysis as well as to demonstrate the appropriate postoperative positions after PPV with gas tamponade for RRDs based on the gas volume and the location of breaks.

## Methods

### Computational eye model

**Figure 1:**
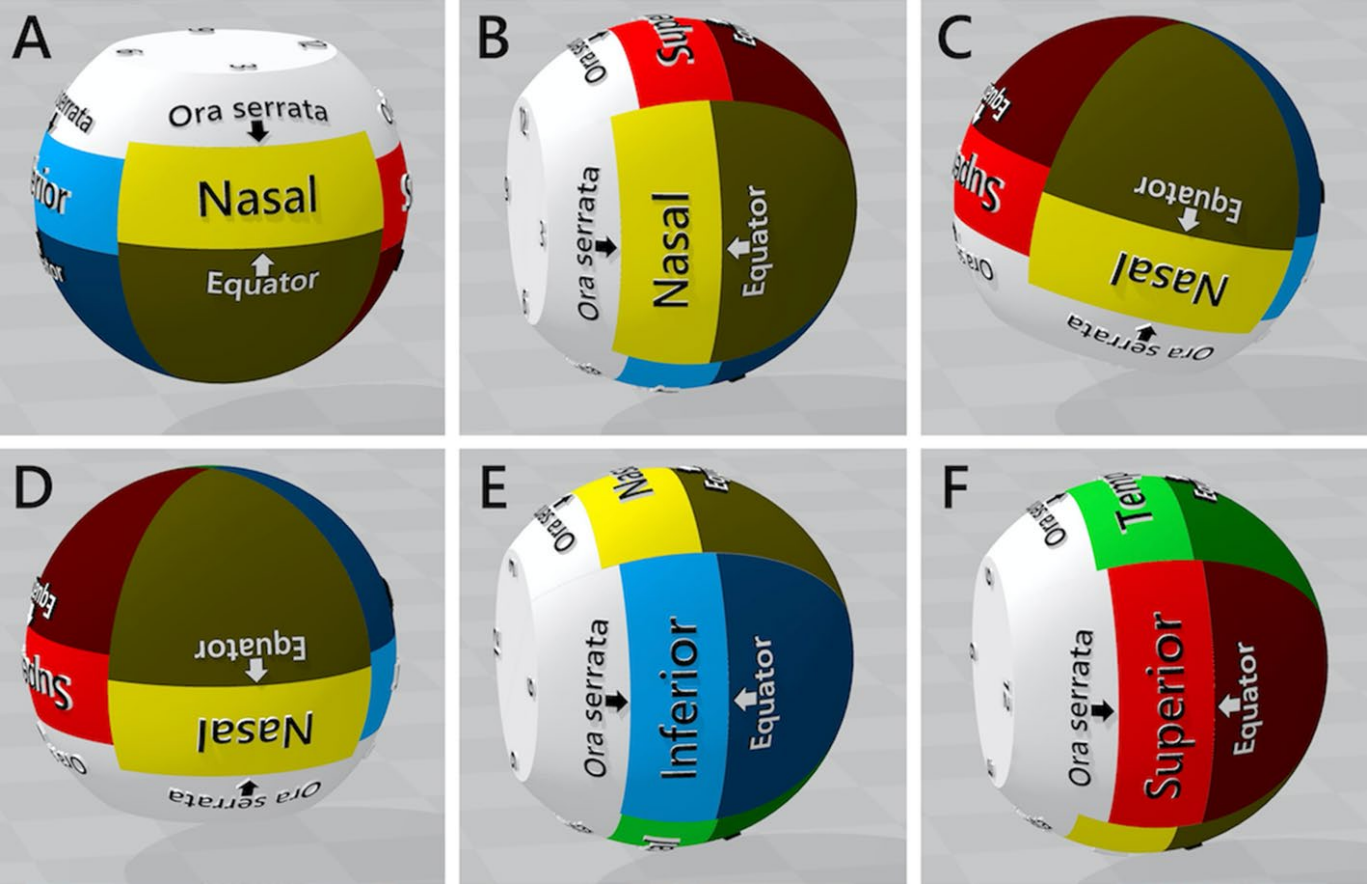
The 24-mm eye model of a pseudophakic eye and resting positions. (**A**) Supine; (**B**) sitting, (**C**) prone with closed eyes, (**D**) prone, (**E**) lower temporal, and (**F**) lower nasal positions.

A computer model of a pseudophakic eye was designed using three-dimensional (3D) modeling software (3D Builder; Microsoft Corporation, WA, USA). The model eye was simplified to a sphere, with the anterior 2.5 mm portion of the sphere removed to best reflect the vitreous cavity of a pseudophakic eye^18^, and separated at 1:30, 4:30, 7:30, and 10:30 into four quadrants (Fig. 1). The majority of human eyes with RRDs have an axial length of > 24 mm, therefore, we used 24-mm, 25-mm, 26-mm, and 31-mm diameter sphere for analysis. The maximum vertical and horizontal linear dimensions of the eyeball have been shown to be linearly related to axial length^19^. Hence, when changing the axial length of the eye, the simplified model with the anterior 2.5 mm part removed was maintained, and its overall diameter was proportionately increased or decreased. We then defined the position of the ora serrata of the superior, nasal, temporal, and inferior retina as per the anatomical differences as 7.4 mm, 5.5 mm, 6.9 mm, and 6.7 mm posterior to the simulated limbus, respectively. Next, we defined the equator, according to the vortex vein ampullae, as 6.5 mm posterior to the ora serrata in each retinal area^20^. Therefore, the anterior retina to the equator, where retinal tears are usually found^21^, was separated into four parts: superior, nasal, inferior, and temporal (Fig. 1).

### Direction of the eye model

Figure 1A–F shows resting positions of the 24 mm eye model, which represents the postoperative body positions. We have previously reported that even if patients superficially maintain a strict prone position with eyes closed, the mean supraduction angle (°) of the eyeball to the perpendicular line is positive (16.1°)^16^. Therefore, six resting positions of the eyeball examined were as follows:

1. Supine: The direction of the eyeball is 90° upward from the horizontal line
2. Sitting: The direction of the eyeball is parallel to the horizontal line
3. Prone with closed eyes: The supraduction angle of the eyeball to the perpendicular line is 16.1°
4. Prone: The direction of the eyeball is 90° downward from the horizontal line
5. Lower temporal: The eyeball was rotated 90° from the sitting position, such that the temporal retina was on the horizon side
6. Lower nasal: The eyeball was rotated 90° from the sitting position, such that the nasal retina was on the horizon side

### Calculating the wettability of the surface

CFD software (PHOENICS; Concentration Heat and Momentum Ltd, London) was used to calculate the wettability of the surface of the eye model using cut cell method, with different gas volumes from 10 to 90%, in increments of 10% to the vitreous cavity. The cut cell method is developed particularly for improving the calculation accuracy of diagonally arranged objects. In this method, the intersection of the outline and mesh of the arranged object are investigated, and the area and volume in which the fluid flows are calculated. When friction occurs, the friction force is given by vector-decomposing the intersection coordinates in the direction of the solid inclination. The water properties were as follows: temperature, 37 °C; density, 998.23 kg/m^3^; kinematic viscosity, 1.006E−6 m^2^/s; and volume expansion rate, 1.18E−4 1/K. We used air properties as the tamponade gas and they were as follows: temperature, 37 °C; density, 1.1892 kg/m^3^; kinematic viscosity, 1.544E−05 m^2^/s; and volume expansion rate, 3.41E−3 1/K. The air–water–retina interface was characterized by a surface tension of 0.072 N/m and a contact angle of 38.8°^5^. A pressure relief point was set in the gas, and the surface wettability of the eye model was calculated as the SURN variable, which represents the volume fraction of the water on the eye model and was shown diagrammatically as surface contour.

### Measurement of gas cover rates of the retina

**Figure 2:**
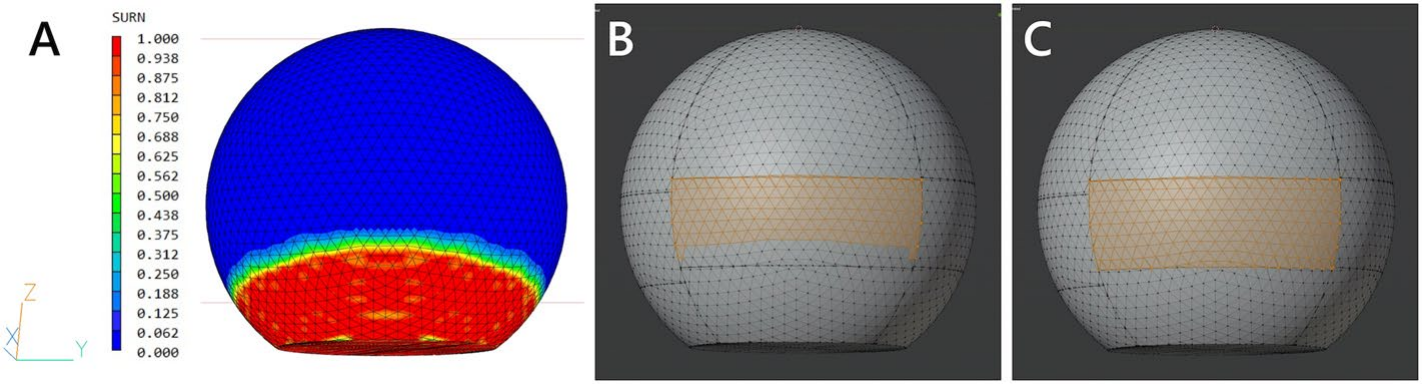
Process of measuring the gas cover rate of the inferior retina in the prone position with closed eyes with 90% gas to the vitreous cavity. (**A**) The surface contour of the SURN variable and wireframe of the eye model are displayed simultaneously. (**B**) The retinal area covered by the gas where the SURN is 0.0, and (**C**) the total retinal area was measured.

Figure 2 shows the process of measuring the gas cover rate. The surface contour of the SURN variable and the wireframe of the eye model are displayed simultaneously, and the gas covered retinal area where the SURN was 0.0 was measured using 3D modeling software (Blender 2.80). The gas cover rate was calculated as (the gas covered area) / (the total area of the retina at each quadrant) × 100% in the supine, sitting, lower nasal, lower temporal, prone with closed eyes, and prone positions.

## Results

### Gas cover rate in each retinal area

#### Superior retina

Table 1 show the gas cover rates of the superior retina of 24-, 25-, 26-, and 31-mm eye model, respectively. In the sitting position, the gas cover rate was 100% until the gas volume was reduced to 10% in all models. In the supine position, the gas cover rate was 100% until the gas volume was reduced to 70% in all models. In the prone with closed eyes position, the gas cover rate was maintained at 100% until the gas volume was reduced to 80% in all models. In the lateral position, the gas cover rate was maintained at 100% until the gas volume was reduced to 90% in all models. In the prone position, the gas cover rate was maintained at 100% until the gas volume was reduced to 90% in 24-mm model and 80% in 25-, 26- and 31-mm models, respectively.

**Table 1:** Gas cover rates of the superior retina of 24-, 25-, 26- and 31-mm eye models.

	Gas volume to the vitreous cavity (%)										
	100	90	80	70	60	50	40	30	20	10	0
**24-mm**											
Supine	100	100	100	100	77	50	24	0	0	0	0
Sitting	100	100	100	100	100	100	100	100	100	100	0
Lateral	100	100	94	66	52	45	34	26	12	4	0
Prone	100	100	89	60	36	11	0	0	0	0	0
Prone with closed eyes	100	100	100	80	67	55	27	10	0	0	0
**25-mm**											
Supine	100	100	100	100	76	66	26	0	0	0	0
Sitting	100	100	100	100	100	100	100	100	100	100	0
Lateral	100	100	83	67	56	44	31	19	8	0	0
Prone	100	100	100	71	38	13	0	0	0	0	0
Prone with closed eyes	100	100	100	94	60	37	15	3	0	0	0
**26-mm**											
Supine	100	100	100	100	80	55	30	0	0	0	0
Sitting	100	100	100	100	100	100	100	100	100	100	0
Lateral	100	100	76	65	51	42	30	18	8	0	0
Prone	100	100	100	71	40	9	0	0	0	0	0
Prone with closed eyes	100	100	100	90	60	38	17	5	0	0	0
**31-mm**											
Supine	100	100	100	100	74	62	27	0	0	0	0
Sitting	100	100	100	100	100	100	100	100	100	100	0
Lateral	100	100	73	62	48	39	29	18	9	0	0
Prone	100	100	100	65	37	8	0	0	0	0	0
Prone with closed eyes	100	100	100	89	59	33	20	6	0	0	0

#### Nasal retina

Table 2 show the gas cover rates of the nasal retina of 24-, 25-, 26-, and 31-mm eye model, respectively. In the lower temporal position, the gas cover rate was 100% until the gas volume was reduced to 10% in all models. In the supine position, the gas cover rate was 100% until the gas volume was reduced to 60% in all models. In the sitting and prone positions, the gas cover rate was maintained at 100% until the gas volume was reduced to 90% in all models. In the prone with closed eyes position, the gas cover rate was < 100%, despite the gas volume being 90%. In the lower nasal position, the gas cover rate was already 0%, despite the gas volume being 90% in all models.

**Table 2:** Gas cover rates of the nasal retina of 24-, 25-, 26- and 31-mm eye models.

	Gas volume to the vitreous cavity (%)										
	100	90	80	70	60	50	40	30	20	10	0
**24-mm**											
Supine	100	100	100	100	100	78	53	29	0	0	0
Sitting	100	100	92	68	51	43	33	26	14	4	0
Lower nasal	100	0	0	0	0	0	0	0	0	0	0
Lower temporal	100	100	100	100	100	100	100	100	100	100	0
Prone	100	100	67	33	9	0	0	0	0	0	0
Prone with closed eyes	100	91	59	11	0	0	0	0	0	0	0
**25-mm**											
Supine	100	100	100	100	100	89	52	25	0	0	0
Sitting	100	100	78	65	54	44	30	17	8	0	0
Lower nasal	100	0	0	0	0	0	0	0	0	0	0
Lower temporal	100	100	100	100	100	100	100	100	100	100	0
Prone	100	100	72	47	14	0	0	0	0	0	0
Prone with closed eyes	100	95	62	37	0	0	0	0	0	0	0
**26-mm**											
Supine	100	100	100	100	100	80	54	30	0	0	0
Sitting	100	100	73	62	49	39	27	16	7	0	0
Lower nasal	100	0	0	0	0	0	0	0	0	0	0
Lower temporal	100	100	100	100	100	100	100	100	100	100	0
Prone	100	100	70	47	18	0	0	0	0	0	0
Prone with closed eyes	100	92	61	34	0	0	0	0	0	0	0
**31-mm**											
Supine	100	100	100	100	100	90	52	24	0	0	0
Sitting	100	100	73	62	49	41	31	18	8	0	0
Lower nasal	100	0	0	0	0	0	0	0	0	0	0
Lower temporal	100	100	100	100	100	100	100	100	100	100	0
Prone	100	100	68	46	15	0	0	0	0	0	0
Prone with closed eyes	100	90	63	38	0	0	0	0	0	0	0

#### Temporal retina

Tables 3 show the gas cover rates of the temporal retina of 24-, 25-, 26-, and 31-mm eye model, respectively. In the lower nasal position, the gas cover rate was 100% until the gas volume was reduced to 10% in all models. In the supine position, the gas cover rate was 100% until the gas volume was reduced to 70%. In the sitting and prone positions, the gas cover rate was maintained at 100% until the gas volume was reduced to 90% in all models. In the prone with closed eyes position, the gas cover rate was < 100%, despite the gas volume being 90% in all models. In the lower temporal position, the gas cover rate was already 0%, despite the gas volume being 90% in all models.

**Table 3:** Gas cover rates of the temporal retina of 24-, 25-, 26- and 31-mm eye models.

	Gas volume to the vitreous cavity (%)										
	100	90	80	70	60	50	40	30	20	10	0
**24-mm**											
Supine	100	100	100	100	87	62	38	11	0	0	0
Sitting	100	100	91	67	51	44	33	26	12	4	0
Lower nasal	100	100	100	100	100	100	100	100	100	100	0
Lower temporal	100	0	0	0	0	0	0	0	0	0	0
Prone	100	100	86	47	25	0	0	0	0	0	0
Prone with closed eyes	100	95	78	38	13	6	0	0	0	0	0
**25-mm**											
Supine	100	100	100	100	87	73	37	11	0	0	0
Sitting	100	100	79	65	54	42	30	19	8	0	0
Lower nasal	100	100	100	100	100	100	100	100	100	100	0
Lower temporal	100	0	0	0	0	0	0	0	0	0	0
Prone	100	100	78	60	22	0	0	0	0	0	0
Prone with closed eyes	100	93	77	59	18	4	0	0	0	0	0
**26-mm**											
Supine	100	100	100	100	87	65	39	13	0	0	0
Sitting	100	100	74	62	49	39	29	19	7	0	0
Lower nasal	100	100	100	100	100	100	100	100	100	100	0
Lower temporal	100	0	0	0	0	0	0	0	0	0	0
Prone	100	100	71	59	24	0	0	0	0	0	0
Prone with closed eyes	100	86	72	54	20	3	0	0	0	0	0
**31-mm**											
Supine	100	100	100	100	86	74	37	15	0	0	0
Sitting	100	100	75	64	50	41	29	17	8	0	0
Lower nasal	100	100	100	100	100	100	100	100	100	100	0
Lower temporal	100	0	0	0	0	0	0	0	0	0	0
Prone	100	100	78	58	20	0	0	0	0	0	0
Prone with closed eyes	100	89	77	58	22	4	0	0	0	0	0

#### Inferior retina

Tables 4 show the gas cover rates of the inferior retina of 24-, 25-, 26-, and 31-mm eye model, respectively. In the supine position, the gas cover rate was 100% until the gas volume was reduced to 70% in all models. In the prone and lateral positions, the gas cover rate was 100% until the gas volume was reduced to 90% in all models. In the prone with closed eyes position, the gas cover rate was already 75% in 24-mm model and < 60% in 25-, 26-, and 31-mm models, respectively, although the gas volume was 90%. In the sitting position, the gas cover rate was already 0%, despite the gas volume being 90% in all models.

**Table 4:** Gas cover rates of the inferior retina of 24-, 25-, 26- and 31-mm eye models.

	Gas volume to the vitreous cavity (%)										
	100	90	80	70	60	50	40	30	20	10	0
**24-mm**											
Supine	100	100	100	100	88	62	39	13	0	0	0
Sitting	100	0	0	0	0	0	0	0	0	0	0
Lateral	100	100	94	67	52	44	33	26	12	4	0
Prone	100	100	86	47	24	0	0	0	0	0	0
Prone with closed eyes	100	75	38	0	0	0	0	0	0	0	0
**25-mm**											
Supine	100	100	100	100	88	74	39	13	0	0	0
Sitting	100	0	0	0	0	0	0	0	0	0	0
Lateral	100	100	79	65	53	43	30	17	8	0	0
Prone	100	100	77	59	22	0	0	0	0	0	0
Prone with closed eyes	100	54	21	0	0	0	0	0	0	0	0
**26-mm**											
Supine	100	100	100	100	88	66	42	15	0	0	0
Sitting	100	0	0	0	0	0	0	0	0	0	0
Lateral	100	100	74	62	50	39	27	16	7	0	0
Prone	100	100	70	58	24	0	0	0	0	0	0
Prone with closed eyes	100	55	21	0	0	0	0	0	0	0	0
**31-mm**											
Supine	100	100	100	100	88	75	38	16	0	0	0
Sitting	100	0	0	0	0	0	0	0	0	0	0
Lateral	100	100	75	64	49	41	29	18	8	0	0
Prone	100	100	78	56	20	0	0	0	0	0	0
Prone with closed eyes	100	57	21	0	0	0	0	0	0	0	0

## Discussion

To the best of our knowledge, this is the first study to demonstrate using fluid analysis to show how the retinal gas cover rate changes due to the intraocular gas volume and positions. Our results demonstrate that if retinal breaks were located anterior to the equator, the prone position may not provide an adequate gas coverage for the retina. In contrast, if breaks are limited to the inferior retina anterior to the equator or if multiple breaks are located in two or more quadrants anterior to the equator, the supine position maintains a 100% gas cover rate in all retinal breaks for longer than other positions. Although the potential postoperative complications caused by the supine position require careful attention, our result may contribute to better surgical outcomes of RRDs, a reduction in the duration of the postoperative prone position and may reduce the patients’ discomfort after PPV with gas tamponade for RRDs.

**Figure 3:**
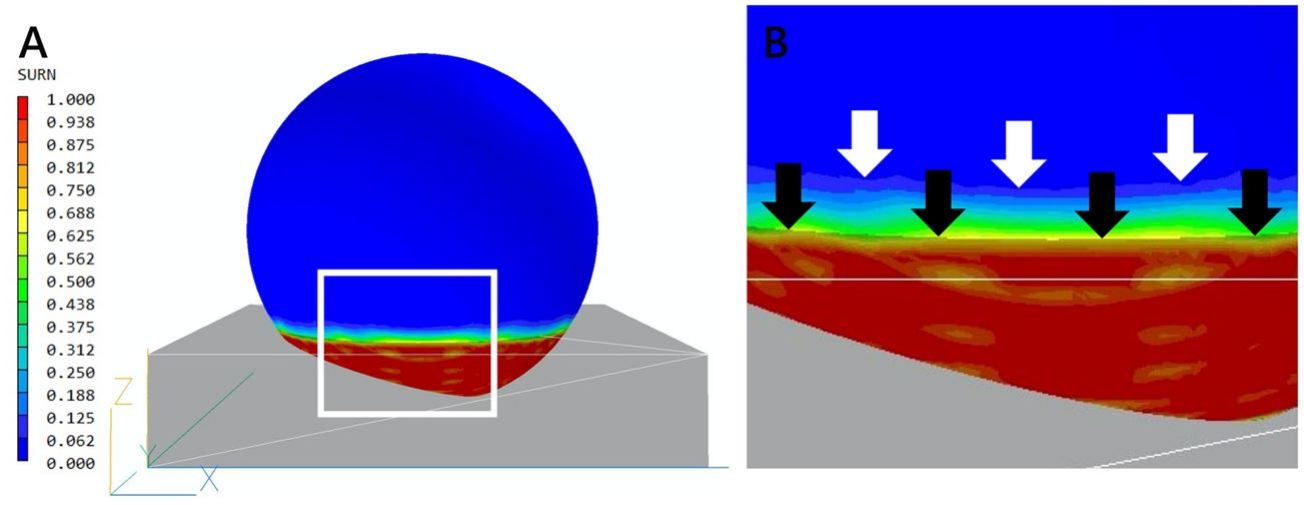
Surface contour in the prone with closed eyes position, with 90% gas to the vitreous cavity. (**A**) From the side view. The gray translucent blockage represents the water object set to have a 10% volume to the vitreous cavity before fluid analysis. The surface contour represents after analysis. (**B**) Enlarged image of (**A**). The black arrows indicate the initial height of the water before analysis, and the white arrows indicate the line where the SURN is 0.0, which indicates complete coverage by the gas after analysis. The existence of surface tension and contact angle cause the line of the SURN = 0.0 to rise above the initial water level, which leads to a reduction in the gas cover rate of the retina.

The contact angle between a given tamponade gas and the retinal surface depends on the interactions of three phases: gas, retina, and fluid. It has been reported that the mean contact angle measured for air bubble against the retina in the fluid was 38.8°^5^. In the current study, fluid analysis, considering the contact angle, and the surface tension allowed the gas cover rates of the retina to be calculated more accurately. The existence of a contact angle and meniscus leads to a reduction in the gas cover rate for a given volume of gas (Fig. 3).

Yoon et al. reported that the adhesive force was transiently reduced after laser photocoagulation of the retina, but increased beyond normal, and remained twice that of normal between 3 days and 4 weeks^22^. These findings indicate that all retinal breaks should be closed with the intraocular gas after PPV with gas tamponade until the strength of the adhesion by photocoagulation sufficiently prevents retinal redetachment. In the current study, all gas cover rates in each quadrant in prone position were < 100% when the gas volume was 80%. Furthermore, we have previously reported that if patients were superficially maintained in a strict prone position, the supraduction angle (°) of the eyeball to the perpendicular line was positive (16.1°; range, 6.3–29.9)^16^. In the current study, in the prone with closed eyes position, the gas cover rates of the nasal and temporal retinas were < 100%, despite the intraocular gas volume being 90%. Surprisingly, the gas cover rate of the inferior retina in the prone with closed eyes position was < 80%, despite the intraocular gas volume being 90%. Martinez-Castillo et al. reported that only 14.4% cases presented with a vitreous cavity that was filled ≥ 90% by air or gas at the 1 and 3 days postoperative days after PPV with air or gas tamponade for RRDs^15^. Therefore, if the location of the breaks are limited to either superior, nasal, or temporal, the most appropriate position is sitting, lower temporal, and lower nasal, respectively. However, in the supine position, the gas cover rates of the superior, nasal, and temporal retina were maintained at 100% until the intraocular gas volume was 70%, 60%, and 70% in all models, respectively. Therefore, the supine position may be appropriate for better gas coverage for the retina as the second choice of the position in cases where the breaks are localized at either the superior, nasal, or temporal retina. In addition, if the breaks are limited to the inferior retina anterior to the equator or if multiple breaks are in two or more quadrants anterior to the equator, the supine position may provide 100% gas cover rate in all retinal breaks for longer than other positions.

The supine position may cause some postoperative complications, such as pupillary block, anterior chamber shallowing, and IOL dislocation/iris capture. Furthermore, Shiragami et al. demonstrated that the immediate prone or facedown position after PPV with gas tamponade may prevent the retina from postoperative retinal translocation if the retinal detachment was large or if macular detachment was present^6^. In contrast, the postoperative facedown position reported for only 2 h prevents retinal translocation after PPV with gas tamponade for RRDs^23^. In addition, Otsuka et al. reported no significant difference in postoperative complications between patients with RRDs in a prone position and those in a prone position on the day of surgery followed by the supine position^24^. Therefore, considering these reports as well as our results, patients with RRDs should take a strict prone or facedown position immediately following PPV with gas tamponade and maintain their position on the day of surgery. This should be followed by maintaining the appropriate position, including supine position as above on the basis of the location of breaks and the intraocular gas volume until the gas volume decreases to 70% in the vitreous cavity.

This study has some limitations. High myopia has been reported to have an irregular shape^25^; however, because this study involves simulation using a 3D eye model, irregular-shaped eyes could not be analyzed. This study did not consider eyeball movement, such as rotation, adduction, and abduction, as the simulation is of a stationary 3D eye model. Moreover, we considered that only one bubble was observed in the vitreous cavity and did not consider the case of multiple bubbles. Often a computer model needs a physical model for verification, which has not been performed in this study. Gas properties used in this study were of air, but clinically, different types and concentrations of gases (SF6, C2F6, and C3F8) other than air are used to treat RRD eyes. This study only considered the location of retinal breaks, not the extent of retinal detachment.

In conclusion, we demonstrated that the supine position may provide 100% gas cover rate for all retinal breaks longer than other positions when the breaks are limited to the inferior retina or multiple breaks are in two or more quadrants anterior to the equator. Although complications occurring due to the supine position should be considered, our result may contribute to better surgical outcomes of RRDs and reduced duration of a strict prone position that leads to reduced patient discomfort after PPV with gas tamponade for RRDs.

## Data Availability

The datasets generated during and/or analyzed during this study are not publicly available but are available from the corresponding author on reasonable request.
